# Different spectrophotometric methods for simultaneous quantitation of Vericiguat and its alkaline degradation product: a comparative study with greenness profile assessment

**DOI:** 10.1038/s41598-023-50097-1

**Published:** 2023-12-27

**Authors:** Doaa M. Mustafa, Nancy Magdy, Noha F. El Azab

**Affiliations:** Department of Pharmaceutical Analytical Chemistry, Faculty of Pharmacy, Ain Shams University, Organization of African Unity Street, Abbassia, Cairo, 11566 Egypt

**Keywords:** Drug discovery, Chemistry

## Abstract

Investigations concerning novel drugs and their induced degradation products are necessary for clinical research and quality control in the pharmaceutical industry. Four spectrophotometric techniques have been performed for simultaneous quantitation of Vericiguat (VER) and its alkali-induced degradation product (ADP) without prior separation. Method A is a dual wavelength method (DW) that estimates the absorbance difference at 314–328 nm, and 246–262 nm for VER and ADP; respectively. Method B uses a ratio difference method (RD) to estimate the ratio spectrum’s amplitude difference (DP_318-342_) and (DP_284-292_) for VER and ADP; respectively. Method C uses a first derivative ratio method (^1^DD) to estimate the peak ratio spectrum amplitude of the first derivative at 318 and 275 nm for VER and ADP; respectively. Method D uses the mean centering of the ratio spectra (MCR) to estimate amplitude values for VER and ADP at 337 and 292 nm; respectively. In a concentration range of 5.00–50.00 µg/mL for VER and 5.00–100.00 µg/mL for ADP, the methods were validated following ICH criteria and utilized to estimate VER in bulk and its dosage form. The methods’ greenness was assessed via three tools: the green analytical procedure index (GAPI), analytical eco-scale, and analytical greenness assessment (AGREE).

## Introduction

Heart failure (HF), also referred to as congestive heart failure, is the severe end stage of several cardiovascular disorders, such as hypertension, coronary heart disease, heart inflammation, and cardiomyopathy. It is a failure to meet the needs of circulation on a systemic level adequately. In short, the heart is unable to keep up with its workload. Heart failure continues to have a high morbidity and mortality incidence on a global scale. It is thought to affect 26 million people worldwide, which raises the expense of healthcare services^1^. HF is frequently categorized into three types according to the left ventricular ejection fraction: (1) heart failure with reduced ejection fraction (HFrEF)-EF ≤ 40%, (2) heart failure with mildly reduced ejection fraction (HFmrEF)-EF ranging from 41 to 49% and (3) heart failure with preserved reduced ejection fraction (HFpEF)-EF ≥ 50%^2^.

Vericiguat (VER), an innovative oral soluble guanylate cyclase (sGC) agonist, has recently been recommended for (HFmrEF)-EF > 45% therapy to lower the need for outpatient intravenous (IV) diuretics and lower the risk of cardiovascular death and hospitalizations associated with heart failure^3,4^. The smooth muscle cells of blood vessels include an enzyme called sGC, which functions as a receptor for nitric oxide (NO). Once NO attaches to sGC, it enhances the generation of the second messenger, guanosine 3′, 5′-cyclic monophosphate (cGMP), which has a crucial function in controlling vascular tone, proliferation, fibrosis, and inflammation. VER activates the NO–sGC–cGMP pathway by directly stimulating sGC receptors and increasing the sGC's sensitivity to intrinsic nitric oxide (NO), regardless of NO availability, which induces the dilation and relaxation of blood vessels^5,6^.

**Figure 1:**
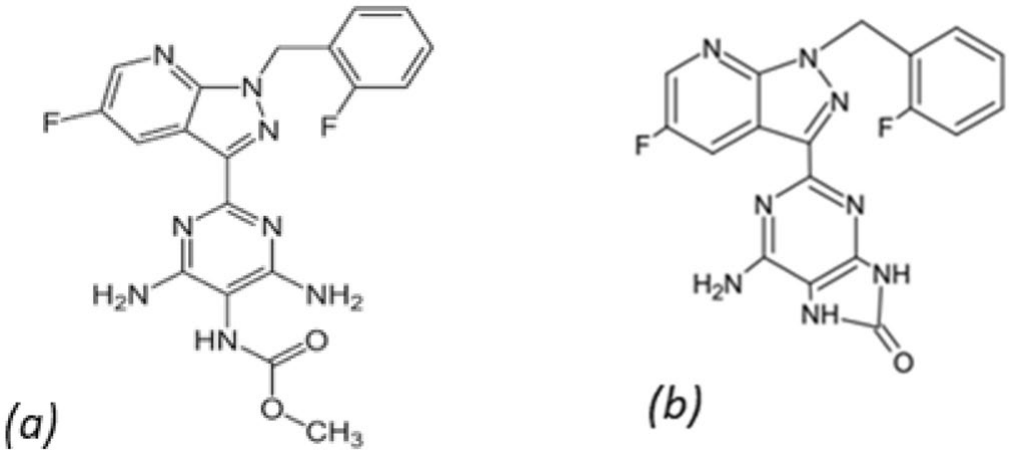
Chemical structure of (**a**) VER, (**b**) ADP.

The IUPAC name of VER is methyl(4,6-diamino-2-(5-fluoro-1-(2-fluorobenzyl)-1H-pyrazolo[3,4-b] pyridin-3-yl) pyrimidin-5-yl) carbamate (Fig. 1a). VER was marketed under Verquvo® trademark by Merck since it was given FDA approval and permission for use throughout the European Union in 2021^7,8^.

The major degradation pathway of VER was found to be alkaline hydrolysis. VER’s alkaline degradation yields amino-2-[4-fluoro-1-(2-fluorobenzyl)-1H-pyrazolo[3,4-b] pyridin-3-yl]-7,9-dihydro-8H-purine-8-one (Fig. 1b). This degradation involves loss of the methoxy group causing carbonyl and amino groups of the pyrimidine ring to be cycled intramolecularly^9^. Hence, it is essential and practical to investigate VER and its alkali-induced degradation product (ADP), which would be significant in the routine analysis of VER for both clinical research and pharmaceutical quality control^10,11^.

The literature survey reveals a stability-indicating HPLC/DAD technique^9^ and a spectrophotometric approach based on the diazo coupling reaction with 8-hydroxyquinoline^12^. Lately, a UPLC-MS/MS method for determining vericiguat in rat plasma has been reported^13^. At this point, no method has been reported for the simultaneous determination of VER and its alkali-induced degradation product using spectrophotometric techniques. Spectrophotometry is still a highly common technique, due to its simplicity and affordability. In addition, the analysis is non-destructive to the sample and relatively rapid compared to other analytical methods. Since spectrophotometry saves time, cost, and effort, it can be regarded as economically efficient. Spectrophotometric techniques are preferred as they overcome the drawbacks of chromatographic methods that are expensive, require high solvent volume, provide moderate throughput, and need highly qualified technicians^14^. The key advantages of the suggested methods over previously spectrophotometric reported method include that they are the first spectrophotometric approaches to determine VER in the presence of its ADP. As a result, they were more comprehensive and specific than the reported procedure. Moreover, the previously reported method was an indirect method based on the diazo coupling reaction with 8-hydroxyquinoline, where the suggested methods were direct methods that avoided the time-consuming reaction steps. Additionally, the reported method can be considered as a sample destructive analysis due to the change of VER’s chemical structure while our methods are non-destructive.

The current work has established and validated four spectrophotometric techniques for the simultaneous quantitation of VER and its alkali-induced degradation product (ADP) in their binary mixtures devoid of requiring any separation processes. The suggested techniques rely on various approaches, including the dual wavelength method (DW)^15^, Ratio difference method (RD)^16–18^, First derivative ratio spectrophotometric method (^1^DD)^19^, and Mean centering of ratio spectra (MCR) method^20,21^. Following the guidelines of the International Conference of Harmonization (ICH), the techniques have been validated to be precise, quick, easy, accurate, and robust regarding use in routine testing and quality control of VER’s dosage form^22–25^. Finally, the proposed approaches' impact on the environment and human health was evaluated quantitatively and qualitatively by employing a variety of green assessment tools.

## Experimental

### Apparatus and software

A UV-1800 PC model, double beam UV–visible spectrophotometer (SHIMADZU, Japan), equipped with a quartz cell size 1 cm, and UV Probe software version 2.50 were employed. The scanning speed is in fast mode with a 0.5 nm interval. The suggested mean centering of ratio spectra (MCR) approach was manipulated via Minitab® version 14.

### Samples

#### Pure sample

Vericiguat with a certified purity of > 98% was imported from China by Chemicalbook Inc.

#### Market sample

Verquvo® tablets which contained 2.5 mg of Vericiguat besides croscarmellose sodium, magnesium stearate, sodium lauryl sulfate, lactose monohydrate, microcrystalline cellulose, and Hypromellose as excipients (Batch number: 88122321, Germany) were obtained from the market.

### Solvents and reagents

HPLC-grade methanol, Hydrochloric acid, and sodium hydroxide were provided by Merck KGaA, Germany.

### Standard solutions

An accurately weighed 100.00 mg of VER was loaded into a 100-mL volumetric flask. Methanol was added, sonicated, and then completed to the appropriate volume to make a stock solution with a 1.00 mg/mL concentration. This solution was diluted using methanol, resulting in a final 100.00 µg/mL working solution.

### Alkaline degradant solution

The alkaline degradant solution was prepared where 50.00 mg of VER was accurately weighed, loaded into a 100-mL stoppered conical flask, and dissolved in 10.00 mL of methanol. 50.00 mL of 1 M NaOH was added and heated at 60 °C in a water bath for 24 h, then cooled, neutralized with 32 mL of 1 M HCl, and evaporated under vacuum. The obtained residue was washed using Milli-Q water (5 times, each 10 mL). After dryness, 10.00 mg of the residue was weighed, transported to a volumetric flask (100-mL), and finally diluted with methanol to prepare a solution of 100.00 µg/mL of the degradant. The purity of the degradant solution was tested by HPLC/DAD (an isocratic elution utilizing water with 0.1% o-Phosphoric acid: acetonitrile (70:30 v/v) over a C_18_ column at 0.80 mL/min flow rate)^9^.

### Procedure

#### Linearity

Different aliquots of VER and ADP were individually transported into two distinct series of volumetric flasks (10-mL) using methanol to complete the final volume. Each aliquot was taken from its corresponding standard working solution (100.00 µg/mL). Absorption spectra were assessed for the resulting solutions at (200–400 nm).

##### Dual wavelength method (DW)

The difference in absorbance at 314–328 nm and 246–262 nm for VER and ADP; respectively versus their respective concentrations, was plotted to establish calibration curves. For each compound, the regression equations were calculated.

##### Ratio difference method (RD)

The ratio spectra were produced by obtaining the absorption spectra of VER and ADP solutions and dividing them by the absorption spectra of 10.00 µg/mL ADP and 25.00 µg/mL VER, as divisors; respectively. Calibration curves were established by plotting the ratio spectrum's amplitude differences at 318–342 nm, and 284–292 nm for VER and ADP; respectively, versus their correlating concentrations. For each compound, the regression equation was calculated.

##### First derivative ratio spectrophotometric method (^1^DD)

The same method as before was used to acquire the ratio spectra. The first derivative of the ratio spectra was calculated utilizing Δλ = 5 nm with a scaling factor equals to 10 for VER, and Δλ = 5 nm and a scaling factor = 30 for ADP. Calibration curves were established by graphing the amplitude values at 318 nm and 275 nm for VER and ADP; respectively, versus the correlating concentrations. The regression equation for each compound was calculated.

##### Mean centering of ratio spectra (MCR) method

To acquire the mean-centered ratio spectra for VER and ADP, Minitab software was used to manipulate the ratio spectra data at ranges of 240–400 nm for VER and 200–350 nm for ADP. Maximum peak amplitudes of the mean centering spectra for VER and ADP; respectively, at 337 nm and 292 nm, were plotted against the correlating concentrations for constructing the calibration curves. For both compounds, the regression equations were calculated.

#### Limit of detection and limit of quantification (LOD and LOQ)

LOD was estimated to be 3.3 σ/S and LOQ equal to 10 σ/S since σ and S represent the standard deviation of intercepts and slope of calibration curves; respectively.

#### Accuracy

Five different concentrations of VER and ADP were determined in three repetitions. Then, recovery % and % RSD were estimated.

#### Precision

In order to assess repeatability and intermediate precision, three various concentrations of VER and ADP were determined in three repetitions on the same day and the three following days. Next, the RSD % values were estimated.

#### Robustness

Three different concentrations of VER and ADP were determined in triplicates using different commercial sources of methanol (Carlo Erba, France), (Advent Biochem, India), and (Thermo Fisher, USA). Then, the mean of recoveries % and RSD % were determined.

#### Analysis of lab-prepared mixtures

Lab-prepared mixtures of various ratios of VER and ADP were created by transferring various aliquots of working solutions of VER and ADP (100.00 µg/mL) into sets of volumetric flasks (10-mL) and filling them with methanol to the desired volume. The methodologies stated above within the “Linearity” section were performed.

#### Analysis of the pharmaceutical dosage form

Fourteen Verquvo® tablets were weighed, ground in a mortar and properly mixed. In a 100-mL volumetric flask, an exact weight of the powder equals to 10.00 mg of VER was transported. The working solution (100.00 µg/mL) was prepared by adding 50.00 mL of methanol, followed by sonication for 20 min, cooling, and volume completion. Finally, the solution was filtered. Various dilutions of the solution were dissolved in methanol to prepare various concentrations of VER and then determined by the correlating regression equation for each previously described approach. At three levels of fortification, the standard addition technique was utilized in order to evaluate the accuracy of the stated methods.

## Results and discussion

Spectrophotometry is a widely used and essential technology in quality control tests throughout the world since the majority of compounds exhibit UV region absorption. However, it can be a challenge to identify the target compounds because molecules that are sensitive to degradation are frequently found alongside their degradation products and may have spectra that are highly overlapped. Direct spectrophotometry failed to estimate VER in its mixture with its alkaline degradation product as their absorption spectra exhibit a significant degree of interference. Thus, our main objective was to utilize certain spectral properties in resolving the mixture’s overlapping spectra by using a variety of resolution approaches. Additionally, spectrophotometry has made significant progress towards green analytical chemistry by utilizing more environmentally friendly solvents. In the current study, four sophisticated and environmentally friendly spectrophotometric techniques were created for the simultaneous quantitative analysis of VER and ADP in their binary mixtures.

### Method optimization

**Figure 2:**
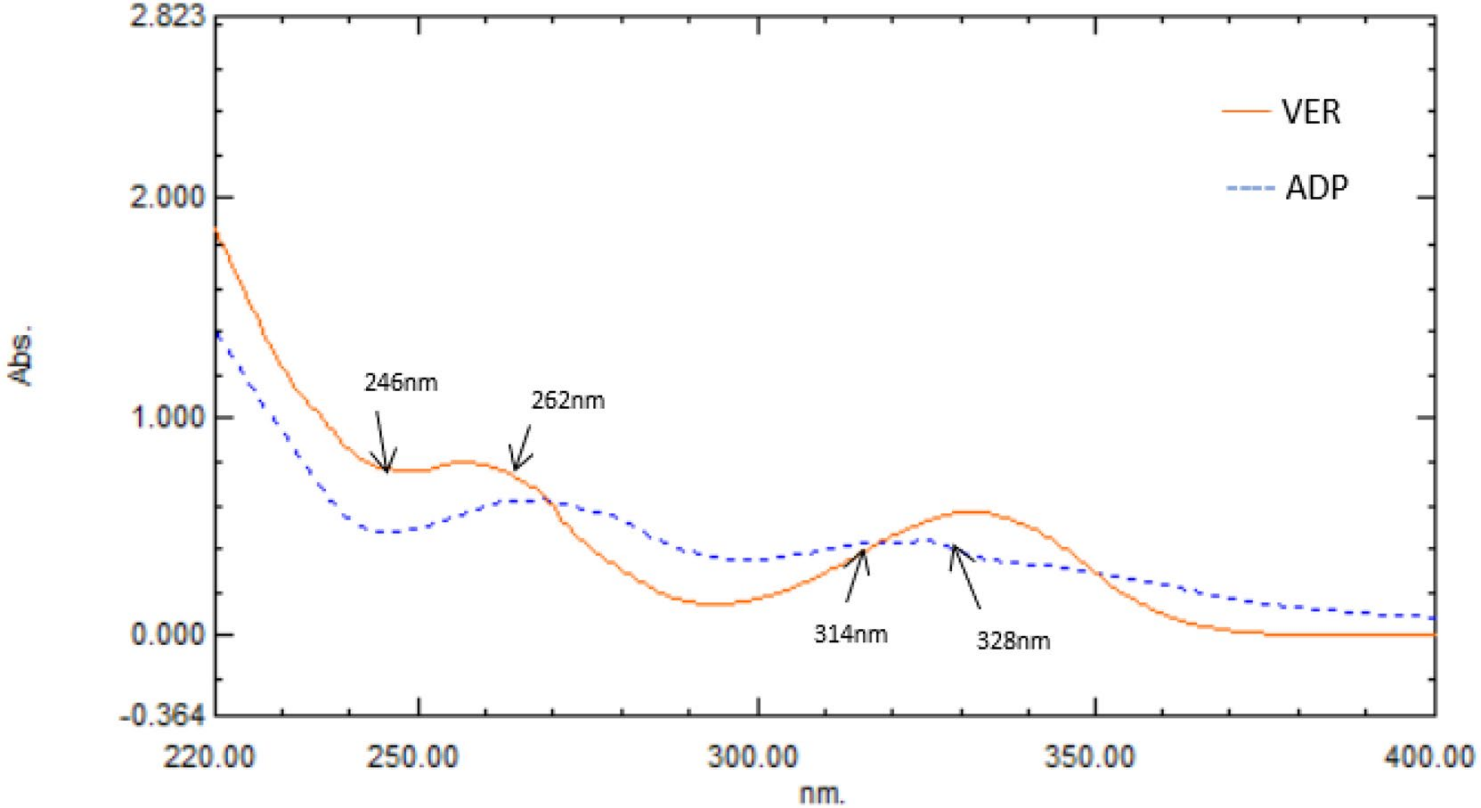
Zero-order absorption spectra of Vericiguat (VER) and its alkaline degradation product (ADP) indicating complete overlap.

Using methanol as a blank, the absorption spectra of 25.00 µg/mL of VER and 50.00 µg/mL ADP were recorded over the 220–400 nm wavelength range. VER is distinguished by two λ_max_ at 256 and 332 nm, while ADP has two characteristic peaks at 269.50 and 325 nm. To choose the appropriate wavelengths for the dual-wavelength spectrophotometric approach, overlay zero-order spectra and spectral characteristics were studied as displayed in Fig. 2.

#### Dual wavelength method (DW)

The UV spectra of VER and ADP, shown in Fig. 2, exhibit extreme overlap, making it very challenging to determine them directly in their binary mixtures. By exploiting their zero-order absorption spectra, the established dual wavelength approach provides a simple way for the selective quantitation of both VER and ADP. The mathematical model of the DW approach was developed and illustrated by Shibata^15^. The principle of this approach is that, regardless of any interfering components, the difference in absorbance between two points on the spectrum is directly proportional to the target component. The prerequisite for this approach is the selection of two wavelengths where the interferant has the same absorbance value and the target component has a notable change in absorbance with concentration. Selection of the suitable wavelengths is crucial; therefore, many different wavelengths were explored for VER and ADP, including 258–277 nm, 285–323 nm, 301–335 nm, and 314–328 nm for VER and 242–256 nm, 318–344 nm, and 323–340 nm for ADP. The optimum selectivity and recovery were observed when the absorbance readings at 314 and 328 nm were the same for ADP. Thus, these two wavelengths were used to determine VER. Likewise, ADP was estimated at 262 and 246 nm where VER showed the same absorbance. The difference between each compound's absorbance values at the chosen wavelengths versus their correlating concentrations was plotted to create calibration curves for VER and ADP. With good correlation coefficients, VER and ADP followed Beer Lambert's law in the concentration ranges of 5.00–50.00 and 5.00–100.00 µg/mL for VER and ADP; respectively as displayed in (Figs. S1 and S2).

This approach is based on the zero-order absorption spectrum, and it was able to resolve the overlapping issue without altering the mixture's initial zero-order absorption spectrum. Saving time and effort, this method also helps retain optimal accuracy by reducing manipulation step errors. The major disadvantage to this approach is that the wavelengths that can be chosen must be those where the interference-causing substance’s absorbance is constant. Due to the poor reproducibility and robustness of the results caused by any small change in the chosen wavelengths, it is necessary to measure the absorbance of the component of interest carefully. In some cases, the small value of amplitude difference between the chosen wavelengths reduces sensitivity.

#### Ratio difference method (RD)

The ratio difference approach is distinguished because of its remarkable simplicity, accuracy, and repeatability. The technique doesn’t need any complicated technology or software programs, and it can solve spectra that have been highly overlapped without any prior separation. The mathematical model of the RD approach was explained by Lotfy et al.^18^. The fundamental principle of the RD is that the target component's concentration, regardless of the interfering component, is directly proportional to the amplitude difference between two points on the mixture's ratio spectrum. Beyond the dual-wavelength approach, the main advantage of such a technique is the total elimination of the interferant in the form of a constant, and any two points’ differences will equal zero, thus, eliminating the need for calibration for critical readings, and leading to extremely reproducible and reliable results. The RD method's limitation is that it is utilized only with binary mixtures, and it is inapplicable when the divisor does not have the maximum sensitivity and the lowest noise.

**Figure 3:**
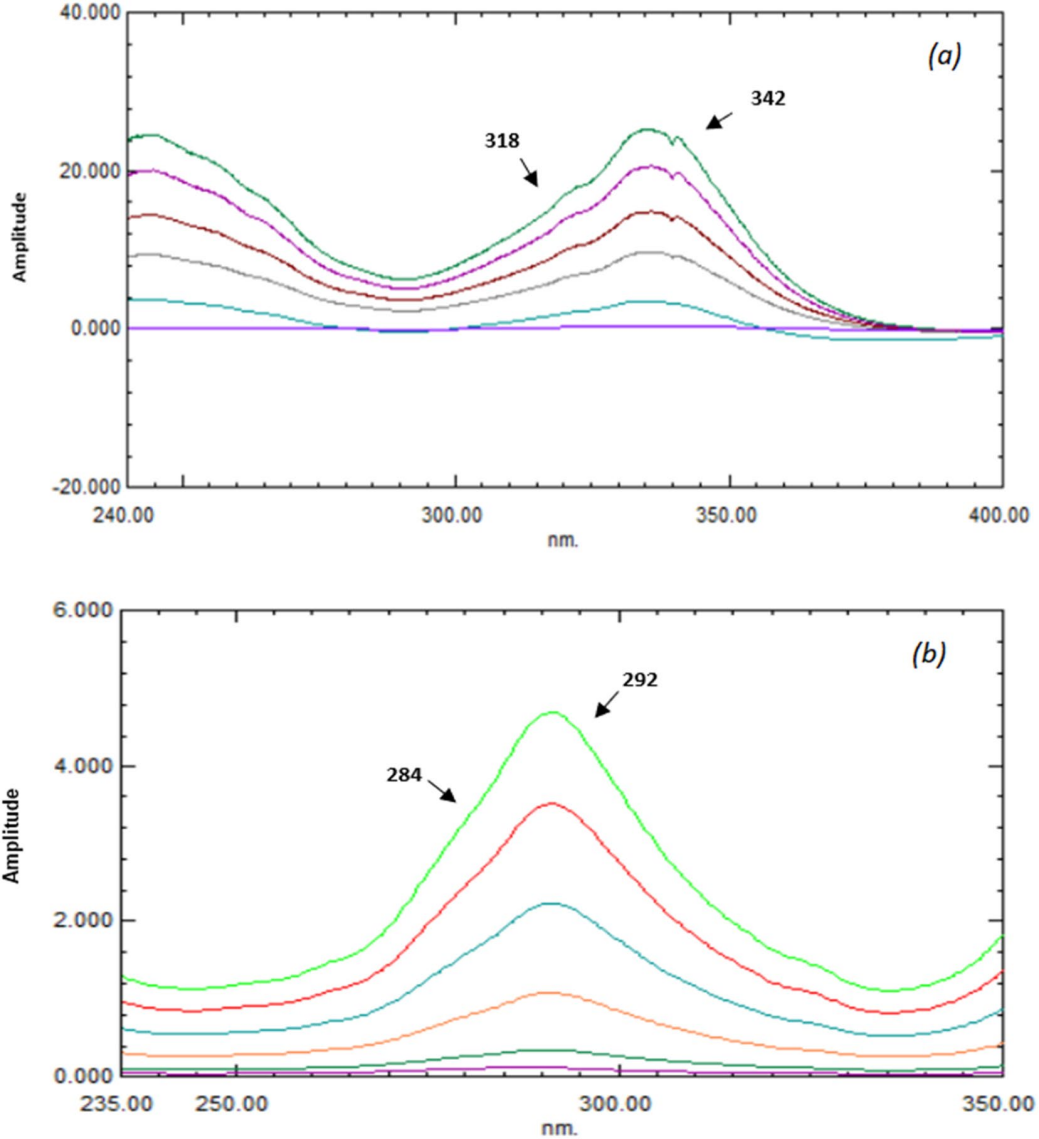
Ratio spectra of (**a**) VER (5–50 μg/mL) using ADP (10 μg/mL) as a divisor, (**b**) ADP (5–100 μg/mL) using VER (25 μg/mL) as a divisor.

The ratio spectra of VER and ADP solutions were recorded using the zero absorption spectra of ADP and VER as a divisor; respectively. The divisor concentration's impact on selectivity was discovered by examining different concentrations of VER (5, 10, 25, and 50 μg/mL) and ADP (5, 10, 25, and 50 μg/mL). Also, various wavelengths were tested, including 320–344, 324–348, and 326–350 nm for VER and 282–294, 286–296, and 288–298 nm for ADP. Regarding sensitivity and selectivity, the best results for quantitation of VER were obtained utilizing 10 μg/mL of ADP spectrum as a divisor at the two chosen wavelengths (318 nm and 342 nm) (Fig. 3a) and 25 μg/mL of VER spectrum as a divisor at (284 nm and 292 nm) for the ADP’s estimation (Fig. 3b). The amplitude differences of ratio spectra between the chosen wavelengths for each compound versus their corresponding concentrations were plotted to create calibration curves for VER and ADP. The regression equations for both compounds were calculated (Figs. S3 and S4).

#### First derivative ratio spectrophotometric method (^1^DD)

**Figure 4:**
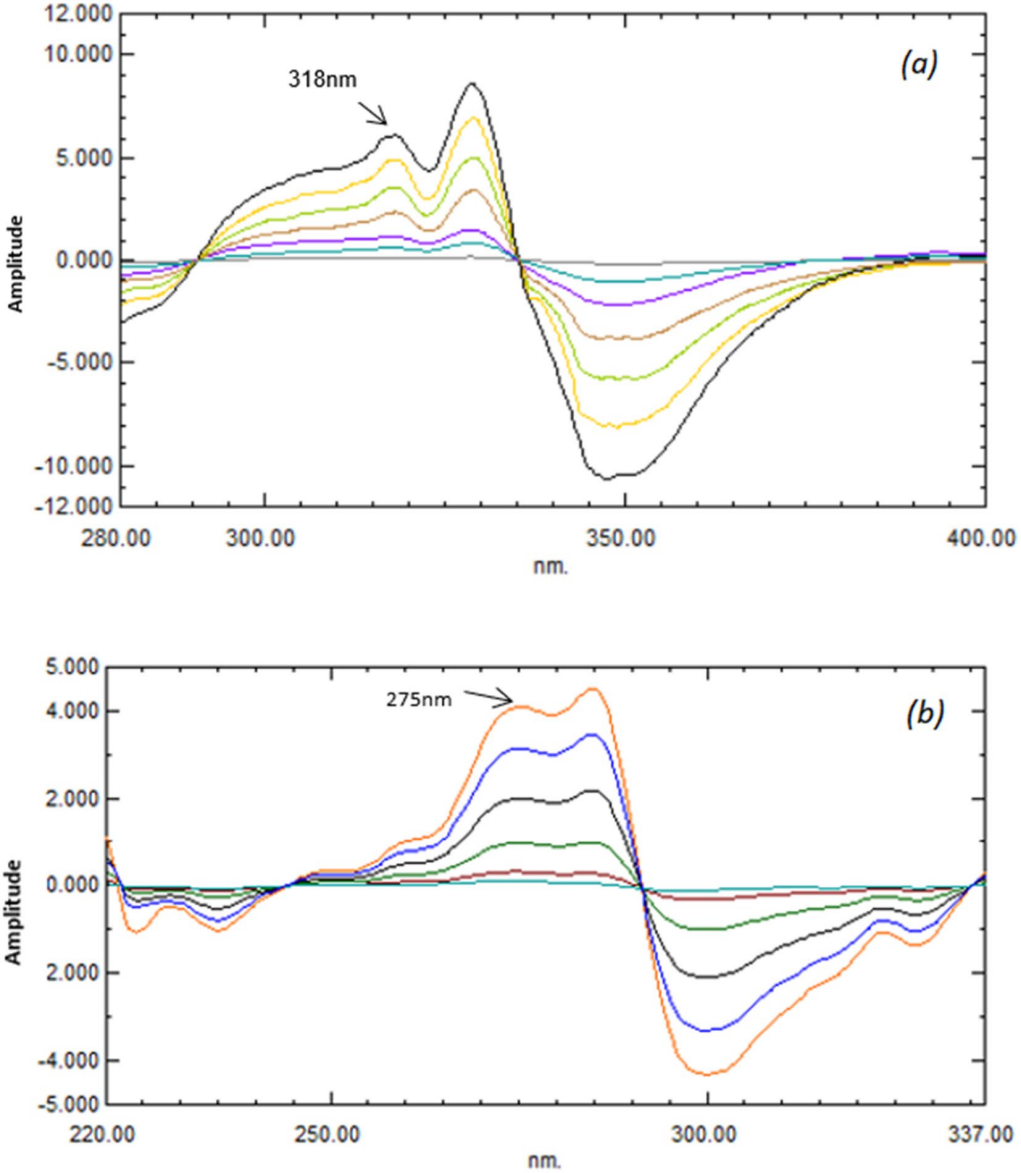
First derivative of ratio spectra of (**a**) VER (5–50 μg/mL) using 10 μg/mL of ADP as a divisor, (**b**) ADP (5–100 μg/mL) using 25 μg/mL of VER as a divisor.

This technique allows the exploitation of the wavelength of the highest sensitivity as the measuring signal, either a minimum or a maximum. The mathematical model of the developed ^1^DD approach was explained by Salinas et al.^19^. The major factors that impact the ratio spectra's shape include wavelength, the standard solution's concentration applied as a divisor, scanning speed, the increment in wavelength that the derivative employs (Δλ), and the smoothing function, all of which have been undertaken through comprehensive testing. The noise level often declines as (Δλ) rises, resulting in fewer prominent fluctuations in the derivative spectrum. The ideal value of (Δλ) should be chosen by considering the noise level, the resolution of the spectrum and the sample concentration, because the spectral resolution is quite poor at too high (Δλ) values. For achieving an acceptable signal-to-noise ratio, several (Δλ) values were evaluated, where 5 nm with scaling factor 10 was found to be the best parameter to determine VER, and 5 nm with scaling factor 30 for ADP determination. Afterwards, the first derivative's peak amplitudes of the ratio spectra were tested at 318, 329, and 347 nm for VER and 275, 285 and 298 nm for ADP determination. The best recovery percentages were obtained at 318 and 275 for VER and ADP; respectively (Fig. 4). The amplitudes of the first derivative ratio spectra at the chosen wavelengths for each compound versus their correlating concentrations were plotted to create calibration curves for VER and ADP. The regression equations for both compounds were calculated (Figs. S5 and S6). The main method's disadvantage is that it needs a lot of regulating processes, such as division and derivative computing, and that it is very sensitive to instrumental factors like slit width and scan rate.

#### Mean centering of ratio spectra (MCR) spectrophotometric method

**Figure 5:**
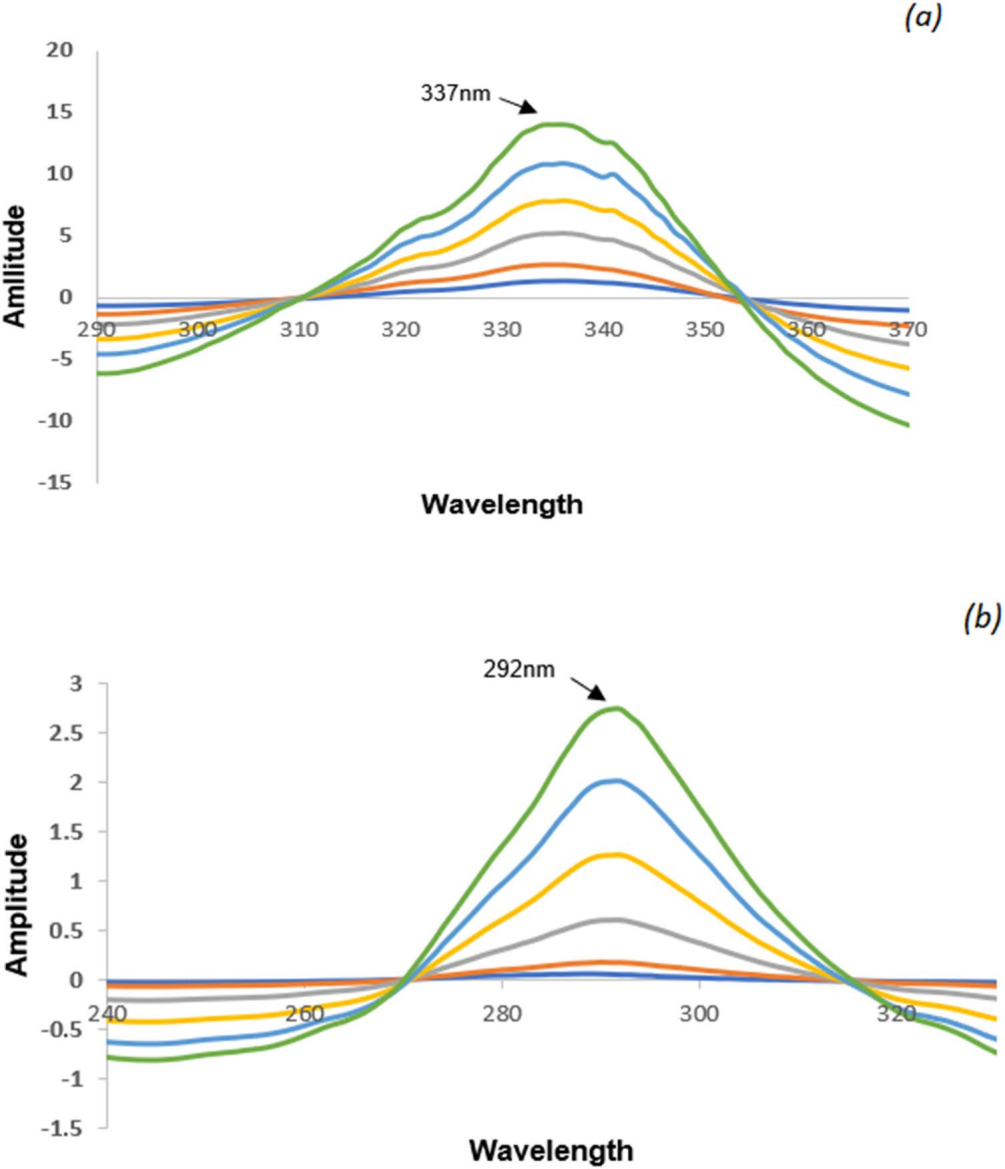
Mean-centered ratio spectra of (**a**) VER (5–50 µg/mL) using ADP (10 µg/mL) as a divisor, (**b**) ADP (5–100 µg/mL) using VER (25 µg/mL) as a divisor.

The mathematical model of the developed MCR approach was illustrated by Afkhami and Bahram^21^. Using this approach, derivative steps are avoided and consequently, the signal-to-noise ratio is enhanced. However, the multiple manipulating steps remained the main disadvantage of this approach. The stored ratio spectra were mean-centered in the range (240–400 nm) and (200–350) for VER and ADP; respectively. The remaining spectra were excluded since they interfered with the linearity of the MC curves. In order to determine the concentrations of VER, the amplitude values of the mean-centered ratio spectra at 337 nm were measured as depicted in Fig. 5a. The amplitude values at 292 nm of the acquired mean-centered ratio spectra were applied for the quantitation of ADP as depicted in Fig. 5b. The regression equation demonstrates the linear correlation between the corresponding concentrations and (MC values) for each compound (Figs. S7 and S8).

### Method validation

Method validation fulfilled ICH recommendation as follows:

#### Linearity

By studying six different concentrations of VER and ADP that ranged between 5.00–50.00 µg/mL and 5.00–100.00 µg/mL; respectively, the techniques were assessed. Three replicates were done for each concentration. The assay was carried out following the above-stated experimental conditions. The calibration equations for the suggested approaches were stated in Table 1 with correlation coefficients greater than 0.9994.

**Table 1:** Linear regression and analytical parameters of the proposed spectrophotometric methods for determination of VER and ADP.

Parameters	(DW)		(RD)		(^1^DD)		(MCR)	
	VER	ADP	VER	ADP	VER	ADP	VER	ADP
Wavelength (nm)	314–328	246–262	318–342	284–292	318	275	337	292
Linearity range (μg/mL)	5.00–50.00	5.00–100.00	5.00–50.00	5.00–100.00	5.00–50.00	5.00–100.00	5.00–50.00	5.00–100.00
Regression equation	Y = 0.0096x−0.0048	Y = 0.0029x−0.0014	Y = 0.1628x−0.4199	Y = 0.0087x−0.0356	Y = 0.1229x−0.0858	Y = 0.0422x−0.0908	Y = 0.2764x−0.2727	Y = 0.0283x−0.1083
Slope	0.0096	0.0029	0.1628	0.0087	0.1229	0.0422	0.2764	0.0283
Intercept	− 0.0048	− 0.0014	− 0.4199	− 0.0356	− 0.0858	− 0.0908	− 0.2727	− 0.1083
Correlation coefficient	0.9995	0.9998	0.9996	0.9998	0.9998	0.9996	0.9996	0.9998
Accuracy (n = 5)	99.72% ± 1.43	100.78% ± 0.89	99.73% ± 1.31	99.97% ± 1.65	99.55% ± 0.87	99.65% ± 1.64	99.69% ± 1.07	100.40% ± 1.76
Precision repeatability (% RSD^a^)	1.06%	1.22%	0.92%	0.90%	0.63%	0.52%	0.34%	0.60%
Intermediate precision (% RSD^b^)	1.40%	1.59%	1.43%	1.74%	1.65%	1.68%	0.59%	1.48%
Robustness (%RSD^c^)	1.44%	1.35%	1.16%	1.21%	0.70%	0.65%	0.75%	0.72%
LOD (μg/mL)	0.921	0.68	0.93	0.87	0.44	0.84	0.35	0.58
LOQ (μg/mL)	2.789	1.99	2.80	2.65	1.44	2.55	1.06	1.75

%RSD^a^, %RSD^b^: intra-day, inter-day respectively (n = 3) of concentrations (5, 25, 50 μg/mL) for VER and ADP. % RSD^c^: relative standard deviation of three replicates of concentrations (5, 25, 50 μg/mL) for VER and ADP using three commercial types of methanol.

#### Limit of detection and limit of quantification (LOD and LOQ)

Limits of detection (LOD) and quantification (LOQ) for both compounds were estimated and stated in Table 1 using the suggested procedures where the MCR showed to be the most sensitive method with LOD of 0.35 and 0.58 for VER and ADP; respectively.

#### Accuracy

Using the described techniques to determine various blind samples of VER and ADP, the concentrations were estimated utilizing the respective regression equations, and the accuracy of the results was validated. Table 1 displays the accuracy as mean % recoveries ranging from 99.55 to 100.78%.

#### Precision

##### Repeatability

Three concentrations of VER and ADP (5, 10, 25 μg/mL) were determined in three replicates intra-daily utilizing the suggested techniques. Acceptable percentage recoveries were achieved with calculated % RSD values ranging from 0.34 to 1.22%, demonstrating the repeatability of the techniques (Table 1).

##### Intermediate precision

The three selected concentrations of VER and ADP were replicated on three successive days. The recorded percentage recoveries with calculated % RSD values ranging from 0.59 to 1.74% were satisfactory as presented in Table 1.

#### Robustness

As the solvent has a significant impact on the spectrophotometric approach, different commercial sources of methanol were examined. It was shown that even slight changes to solvent quality had a negligible impact on the efficacy of the suggested methods. The estimated %RSD values for the suggested methods ranged from 0.65 to 1.44%. Accordingly, the techniques were found to be quite robust with satisfactory % RSD values as established in Table 1.

#### Specificity

Specificity of the techniques was acquired via the determination of various lab-prepared mixtures of VER and ADP within respective linearity ranges. The mean % recoveries for VER ranged from 99.37 to 100.11%, and 99.12–100.35% for ADP. The estimated % RSD values for the assay findings in Table 2 were less than 2%, indicating the specificity of the proposed methods.

**Table 2:** Determination of VER and ADP in laboratory-prepared mixtures by the proposed spectrophotometric methods.

Mix ratio VER/ADP	(DW)		(RD)		(^1^DD)		(MCR)	
	VER	ADP	VER	ADP	VER	ADP	VER	ADP
1:1	99.67%	99.64%	101.82%	101.15%	99.20%	100.39%	99.64%	98.15%
1:2	100.33%	98.76%	99.83%	100.79%	99.55%	100.11%	98.68%	99.78%
2:1	98.34%	98.91%	101.55%	99.23%	99.95%	100.88%	99.80%	99.02%
1:3	98.91%	99.30%	99.67%	100.89%	100.42%	99.93%	100.15%	98.99%
3:1	99.61%	99.28%	98.99%	100.96%	99.91%	100.79%	99.11%	99.67%
3:5	99.66%	98.31%	98.27%	98.57%	99.45%	99.61%	99.17%	99.96%
5:3	99.43%	99.64%	100.63%	100.31%	99.47%	100.74%	99.03%	100.57%
Mean	99.42%	99.12%	100.11%	100.27%	99.71%	100.35%	99.37%	99.45%
± SD	0.63	0.49	1.30	0.99	0.41	0.48	0.51	0.79

### Analysis of VER in Verquvo ® tablets

The described method can successfully determine VER in its commercial dosage form. The drug’s findings were satisfactory and in alignment with the concentration described on the label, where mean % recoveries ± SD were 99.28% ± 0.16, 99.98% ± 0.27, 98.93% ± 0.31, and 98.92% ± 0.15 for DW, RD, ^1^DD, and MCR methods; respectively. The estimated RSD (%) for the assay findings in Table 3 was less than 2%, indicating the high repeatability and accuracy of the proposed method. The interference of excipients in the formulations was investigated using the standard addition method for the pharmaceutical formulation, where mean % recoveries ranged from 99.15 to 100.30% for the proposed methods. As indicated in Table 3, good accuracy confirmed that the pharmaceutical formulation’s excipients were not interfering with the analysis of these substances.

**Table 3:** Determination of VER determination in Verquvo ® tablet by the proposed spectrophotometric methods and application of standard addition technique.

Verquvo ® tablet	(DW)		(RD)		(^1^DD)		(MCR)	
	Found (mg/tablet)	Found %, ± SD^a^	Found (mg/tablet)	Found %, ± SD^a^	Found (mg/tablet)	Found %, ± SD^a^	Found (mg/tablet)	Found %, ± SD^a^
Claimed 2.5 mg/tablet	2.48	99.28% ± 0.16	2.49	99.98% ± 0.27	2.47	98.93% ± 0.31	2.47	98.92% ± 0.15

Standard addition technique								
Taken (mg/mL)	Added (mg/mL)	(DW) Found%	(RD) Found%	(^1^DD) Found%	(MCR) Found%			
10	10 15 20	99.35% 99.71% 98.39%	100.18% 99.70% 101.02%	99.89% 100.27% 98.95%	99.36% 98.59% 99.65%			
Mean ± SD^b^		99.15% ± 0.68	100.30 ± 0.99	99.70% ± 0.69	99.20% ± 0.62			

SD^a^, SD^b^: Standard deviation of VER found %(n = 6), Standard deviation of standard addition at three fortification levels (10, 15, 20 μg/mL) for VER (n = 9); respectively.

### Statistical analysis

To evaluate the practical application of the suggested techniques, the results for determining pure samples of Vericiguat were compared statistically to reported approaches. By setting the p-value at 0.05, it was noticed that the computed student t- and F-values were lower than their corresponding tabulated ones, demonstrating that there was no apparent distinction between the suggested and published HPLC procedure. The findings of the comparative statistical study were represented in Table 4. Moreover, one-way ANOVA was used to compare between and within the four suggested methods, and while the F_cal_ value is smaller than the F_tab_, no significant variations were observed between or within them. Table 5 depicts the one-way ANOVA findings.

**Table 4:** Statistical comparison of the results obtained by the proposed spectrophotometric methods and reported HPLC method for the determination of Vericiguat.

Parameter	(DW)	(RD)	(^1^DD)	(MCR)	Reported method^9^
Mean	99.71	99.73	99.55	99.69	99.71
SD	1.43	1.31	0.87	1.07	1.15
Variance	2.05	1.72	0.75	1.15	1.33
n	5	5	5	5	6
Student’s t-test	0.99 (2.31)	0.98 (2.31)	0.80 (2.26)	0.98 (2.26)	–
F value	1.54 (5.19)	1.29 (5.19)	1.77 (6.26)	1.15 (6.26)	–

At P = 0.05, the values in brackets correspond to the tabulated values.

**Table 5:** One-way ANOVA test obtained for the four proposed spectrophotometric methods for determination of VER.

Source of variation	Sum of square	Degree of freedom	Mean squares	F_cal_	P-value	F_tab_
Between groups	0.10	3	0.03	0.02	0.99	3.23887
Within groups	22.69	16	1.42	–	–	–
Total	22.79	19	–	–	–	–

### Greenness profile assessment

In chemical labs, the idea of green chemistry has gained widespread support to promote sustainable development^26–30^. Specialized assessment tools are stated to accurately estimate the environmental impact of chemical operations^31^. The eco-friendly profile of the proposed UV spectroscopic methods was evaluated via three different greenness evaluation methods. The first method is called eco-scale, and it uses reagents and instruments that fail to adhere to the idea of an ideal green analysis such as penalty points (PP)^32^. By taking into consideration the quantity and hazards of each reagent, the penalty point of the reagent parameter can be determined. On the other hand, the instrument parameter's penalty point is calculated based on the amount of energy consumed by the instrument during operation, the occupational hazard caused by the apparatus, and the amount of waste produced by the device. To estimate the eco-scale of the procedure, penalty points (pp) were calculated and subtracted from 100. The proposed techniques had total penalty points of 9, where methanol taking 6 PP (2 danger signal wards, 3 hazard pictograms, and less than 10 mL of solvent), and the instrument taking 3PP (≥ 0.1 kWh per sample, analytical process hermetization, and waste volume from 1 to 10 mL). As a result, the suggested spectroscopic methods scored 91 on the eco-scale, showing their greenness (Table 6). The green Analytical Procedure Index (GAPI) tool, however, was created as a result of the analytical eco-scale that focused only on the final analytical step while neglecting the other analytical technique phases. In addition to offering an integrated semi-quantitative metric for the entire analytical process, GAPI also offers a five-pentagram design with three-color coding to make visual comparison easier. A total of fifteen parameters were examined, and those with a mild, moderate, or severe environmental impact were given the color green, yellow, or red, accordingly^33^. The suggested techniques were observed to have seven green, and only one red zone (Table 6). Afterward, adopting the recently released AGREE tool, the greenness profile of the suggested methods was evaluated. It is software that generates a colorful pictogram divided into twelve fragments that represent the twelve principles of green analytical chemistry. According to its environmental impact, each component is colored from dark green to dark red, and the total evaluation score is displayed in the center of the circular pictogram^34,35^. The obtained AGREE pictogram, which had a score of 0.68, is displayed in Table 6. Briefly observed, the excellent analytical eco-score, good AGREE score, and prevalence of green color in GAPI pictograms demonstrate the greenness of the suggested procedures.

**Table 6:** Greenness profile assessment of the proposed spectrophotometric methods utilizing different metrics.

Eco-Scale assessment		GAPI tool	AGREE tool
Reagents Methanol^a^	Penalty points 6		
Instruments Energy consumption Occupational hazards Waste	0 0 3		
Total penalty points	Ʃ9		
Analytical Eco scale score^b^	91 Excellent green analysis		

^a^Methanol is labeled a signal word ‘‘danger” with three pictograms and the used volume per sample analysis is < 10 mL (sample cuvette capacity).

^b^ If the score is > 75, it represents excellent green analysis. If the score is > 50, it indicates acceptable green analysis. If the score is > 50, it indicates an unsatisfactory green analysis.

## Conclusions

Despite the total overlapping between the spectra of the two compounds, four suggested spectrophotometric techniques were utilized for accurate and precise quantitation of Vericiguat and its alkaline degradation product in their mixtures without the requirement of prior separation. By using the standard addition technique, the suggested techniques were validated following ICH criteria and used for the assay of VER in Verquvo® tablets. A statistical comparison of the suggested methods and the published HPLC methods revealed insignificant differences in terms of precision and accuracy. Additionally, the most accepted and comprehensive metrics, including analytical eco-scale, GAPI, and AGREE metrics, were implemented to fully guarantee the greenness profile of the suggested approaches. The remarkable simplicity, accuracy, and robustness of the proposed methods, as well as their satisfactory greenness profile, can promote their use in routine analysis and quality control.

### Supplementary Information

Supplementary Figures.

## Data Availability

The datasets generated and analyzed during the current study are available from the corresponding author upon reasonable request.
