# Supplementary material for: Different spectrophotometric methods for simultaneous quantitation of Vericiguat and its alkaline degradation product: a comparative study with greenness profile assessment
Source: Sci Rep. 2023 Dec 27;13:23077. doi: 10.1038/s41598-023-50097-1 (PMC10754859; doi:10.1038/s41598-023-50097-1)

**Supplementary file for**

**Different spectrophotometric methods for simultaneous quantitation of Vericiguat and its alkaline degradation product****[; A comparative study](https://www.sciencedirect.com/science/article/pii/S0026265X2300276X) with greenness profile assessment**

**Doaa M. Mustafa^*^, Nancy Magdy and Noha F. El Azab**

Department of Pharmaceutical Analytical Chemistry, Faculty of Pharmacy, Ain Shams University, Organization of African unity street, Abbassia, Cairo, Egypt, 11566

^*^Corresponding author E-mail: [doaamustafa693@gmail.com](mailto:doaamustafa693@gmail.com)

**Figure S1:** Linearity curve for VER determination by plotting absorbance difference between 314- 328 nm versus corresponding concentration in the range (5.00-50.00µg/mL).


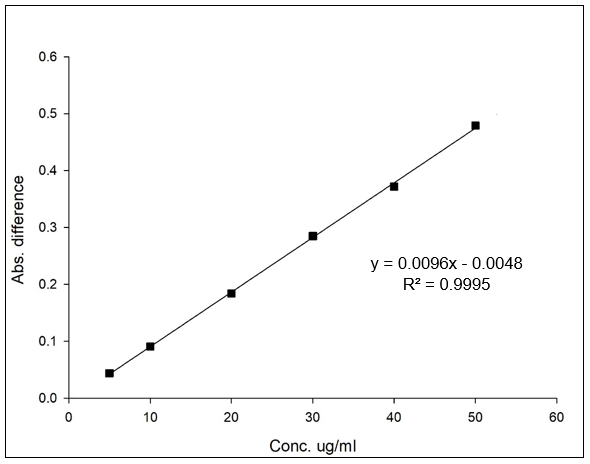


**Figure S2:** Linearity curve for ADP determination by plotting absorbance difference between 246- 262 nm versus corresponding concentration in the range (5.00-100.00 µg/mL).


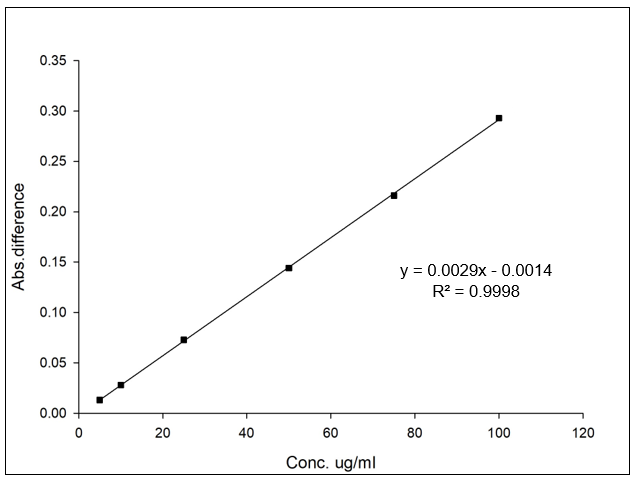


**Figure S3:** Linearity curve for VER determination by plotting amplitudes differences of ratio spectra between 318- 342 nm versus corresponding concentration in the range (5.00-50.00 µg/mL).


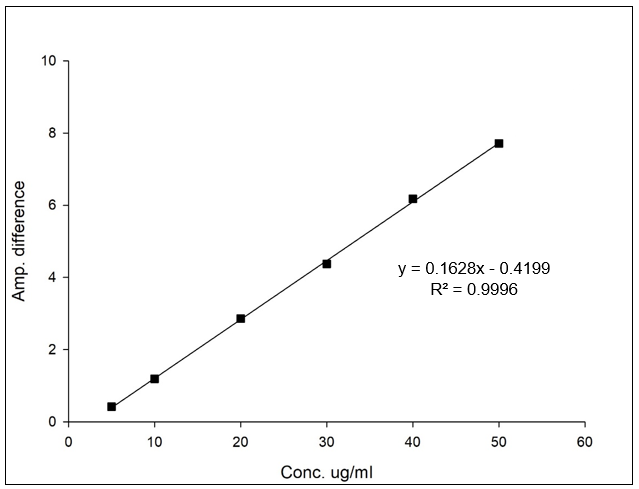


**Figure S4:** Linearity curve for ADP determination by plotting amplitudes differences of ratio spectra between 284-292 nm versus corresponding concentration in the range (5.00-100.00 µg/mL).


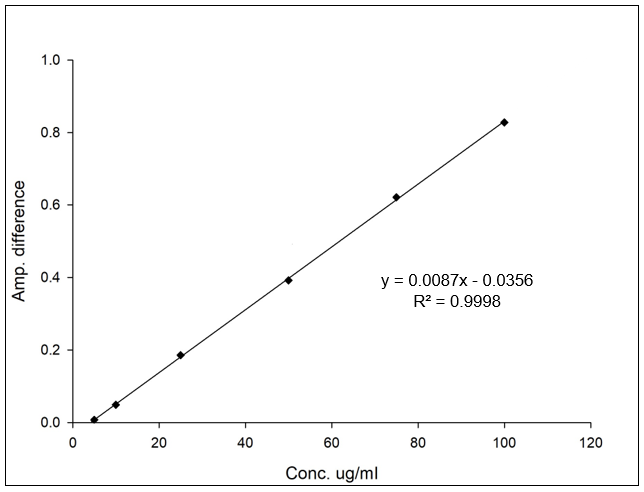


**Figure S5:** Linearity curve for VER determination by plotting amplitudes of the first derivative of ratio spectra at 318 nm versus corresponding concentration in the range (5.00-50.00 µg/mL).


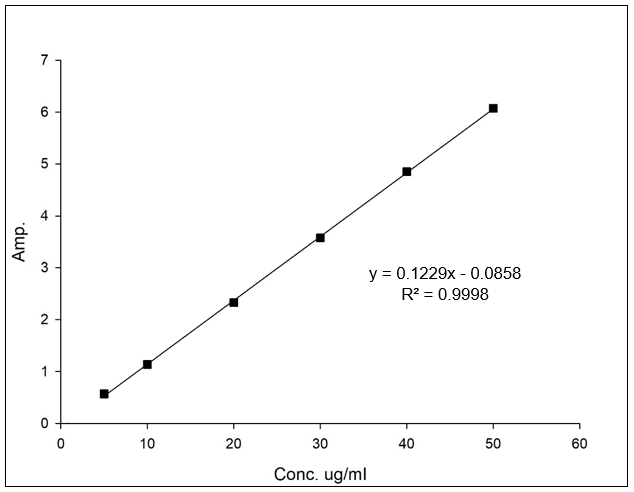


**Figure S6:** Linearity curve for ADP determination by plotting amplitudes of the first derivative of ratio spectra at 275 nm versus corresponding concentration in the range (5.00-100.00 µg/mL).


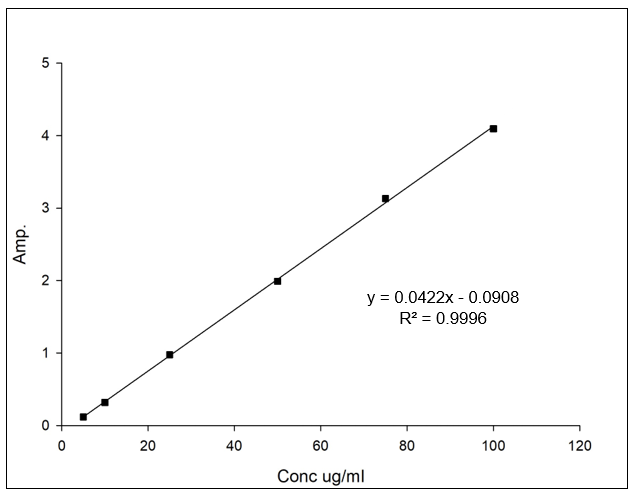


**Figure S7:** Linearity curve for VER determination by plotting amplitudes of the mean-centered ratio spectra at 337 nm versus corresponding concentration in the range (5.00-50.00 µg/mL).


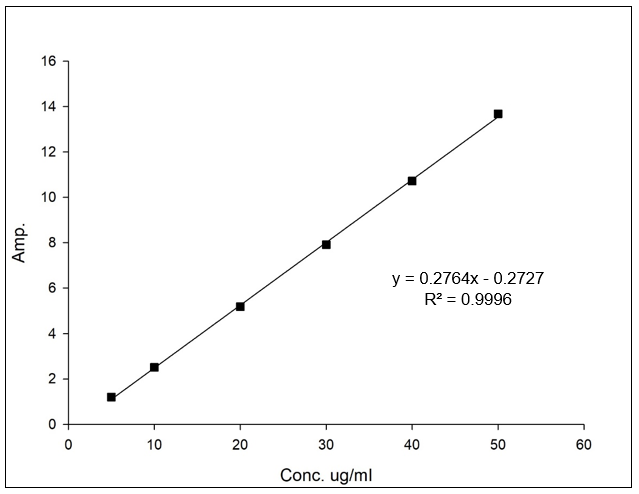


**Figure S8:** Linearity curve for ADP determination by plotting amplitudes of the mean-centered ratio spectra at 292 nm versus corresponding concentration in the range (5.00-100.00 µg/mL).


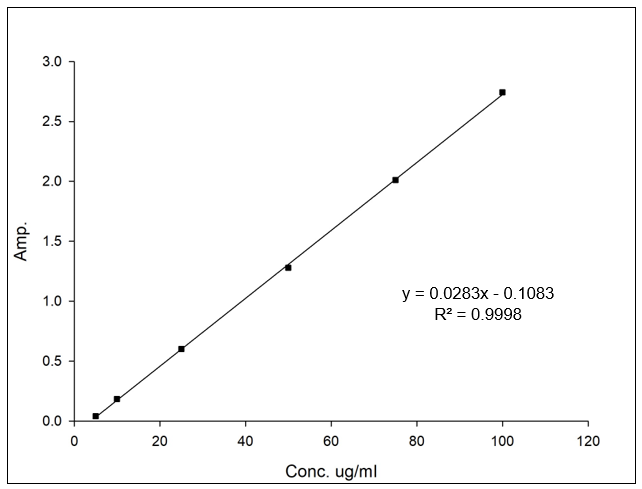

Supplement: Supplementary file 1 — Supplementary Figures. [file 41598_2023_50097_MOESM1_ESM.docx]
